# Recent advances in understanding and managing acute pancreatitis

**DOI:** 10.12688/f1000research.14244.2

**Published:** 2019-01-10

**Authors:** Amar Mandalia, Erik-Jan Wamsteker, Matthew J. DiMagno

**Affiliations:** 1 Division of Gastroenterology and Hepatology, University of Michigan School of Medicine, Ann Arbor, MI, 48109, USA

**Keywords:** Acute pancreatitis, alcohol, gallstones, smoking, cannabis, goal directed fluid therapy, feeding, cholecystectomy, ercp

## Abstract

This review highlights advances made in recent years in the diagnosis and management of acute pancreatitis (AP). We focus on epidemiological, clinical, and management aspects of AP. Additionally, we discuss the role of using risk stratification tools to guide clinical decision making. The majority of patients suffer from mild AP, and only a subset develop moderately severe AP, defined as a pancreatic local complication, or severe AP, defined as persistent organ failure. In mild AP, management typically involves diagnostic evaluation and supportive care resulting usually in a short hospital length of stay (LOS). In severe AP, a multidisciplinary approach is warranted to minimize morbidity and mortality over the course of a protracted hospital LOS. Based on evidence from guideline recommendations, we discuss five treatment interventions, including intravenous fluid resuscitation, feeding, prophylactic antibiotics, probiotics, and timing of endoscopic retrograde cholangiopancreatography (ERCP) in acute biliary pancreatitis. This review also highlights the importance of preventive interventions to reduce hospital readmission or prevent pancreatitis, including alcohol and smoking cessation, same-admission cholecystectomy for acute biliary pancreatitis, and chemoprevention and fluid administration for post-ERCP pancreatitis. Our review aims to consolidate guideline recommendations and high-quality studies published in recent years to guide the management of AP and highlight areas in need of research.

## Introduction

Acute pancreatitis (AP), especially severe cases, is a major clinical and financial burden in the United States. In 2012, AP was the single most common specific gastrointestinal diagnosis for inpatient hospitalization, and annual costs were about $2.6 billion ^1^. Several major clinical guidelines provide evidence-based recommendations for the clinical management decisions in AP, including those from the American College of Gastroenterology (ACG) (2013) ^2^, the American Gastroenterological Association (AGA) (2018) ^3, 4^, and the International Association of Pancreatology (IAP) (2013) ^5^. In this update on AP, we reference recent literature and guideline recommendations focused on epidemiology, risk factors, etiology, diagnosis, risk stratification, and recent advances in the early medical management of AP. Regarding the latter, we review five treatment interventions (intravenous fluid resuscitation, feeding, prophylactic antibiotics, probiotics, and timing of endoscopic retrograde cholangiopancreatography [ERCP] in acute biliary pancreatitis) and four preventive interventions (alcohol and smoking cessation, same-admission cholecystectomy for acute biliary pancreatitis, and chemoprevention and fluid administration for post-ERCP pancreatitis, or PEP). Important management topics not discussed in this review include critical care management in AP (for example, abdominal compartment syndrome ^6^) and late decision making for pancreatitis complicated by infected necrosis, which involves source control with antibiotics and step-up therapy using a combination of percutaneous drainage, endoscopic management, and surgical intervention ^7– 10^.

## Recent advances in epidemiology and evaluation of acute pancreatitis

### Epidemiology

Recent epidemiologic studies show conflicting trends for AP. According to Sellers *et al*., the incidence of AP has decreased in adults from 2007 to 2014 in the United States ^11^. However, data are limited to patients having insurance. In contrast, Krishna *et al*. provide evidence that the incidence of AP in the United States is increasing ^12^, similar to trends over the last few decades. Krishna *et al*. ^12^ extracted data from the Nationwide Inpatient Sample (NIS) and compared two time periods: 2009–2012 (n = 1,070,792) and 2002–2005 (n = 945,253). The major finding was that hospitalizations for AP increased 13.3% ( *p* <0.001). This observed increase is problematic, however, because the analysis did not exclude patients with chronic pancreatitis (CP). The prevalence of CP increased 96%, and CP was the most important independent variable associated with AP hospitalizations between 2009 and 2012 (odds ratio [OR] 35.02, 95% confidence interval [CI] 33.94–36.14). Because smoking was one of three independent predictors for increased risk of CP, it is not surprising that the prevalence of both smoking and CP each increased about 100%. The changes in the odds of AP hospitalization due to other variables were less pronounced from 2009 to 2012 compared with 2002 to 2005: odds (95% CI) decreased for gallstone-related disease—29.85 (33.94–36.14) versus 36.37 (35.32–37.46)—and to a lesser degree for alcohol-related, hyperlipidemia, and diabetes ^12^. Unexpectedly, morbid obesity had a reduced odds of AP that increased modestly over time ^12^. Pancreatic neoplasm had an increased but unchanged odds of AP over time ^12^.

Hospital length of stay (LOS), costs, and mortality declined in 2009–2013 compared with 2002–2005. Mean hospital LOS decreased 0.78 days (−0.85 to −0.70; *p* <0.001), mean hospital costs fell $573 per admission (−$869 to −$277; *p* <0.001), and AP-related mortality declined 30% (3,749 [1.62%] to 2,130 [0.79%]; *p* <0.001). Brown *et al*. attributed these improved outcomes to several potential factors, including routine use of risk stratification tools, increased efficacy of diagnostic tools, and expedited triage of moderate to severe cases to aggressive management in intensive care units ^13^. Multiple studies have also reported a decrease in mortality related to AP ^13– 15^. In support of the possibility that improved management is responsible for decreased mortality, a retrospective study by Agarwal *et al*. demonstrated a decrease in mortality at a tertiary care center in India, despite referral of an increased proportion of patients with organ failure and infected pancreatic necrosis ^16^.

Mortality rates are similar between several etiologies of AP, including gallstone-related and alcohol-induced AP ^17^ and hypertriglyceridemia and alcohol-induced AP ^18^. Persistent organ failure (POF) is defined as organ failure lasting more than 48 hours and is the major cause of death in AP. Additional factors associated with increased mortality include diabetes mellitus ^19^, hospital-acquired infection ^20^, and advanced age (≥70) ^21^. To investigate the controversial association between obesity and mortality in patients with AP, Krishna *et al*. used data from the NIS (2007–2011) to perform propensity score-matched analyses to compare outcomes for adult inpatients with AP with and without morbid obesity ^22^. Morbid obesity was associated with higher frequencies of acute kidney injury (10.8% versus 8.2%; *p* <0.001) and respiratory failure (7.9% versus 6.4%; *p* <0.001) and higher odds of mortality (OR 1.6, 95% CI 1.2–2.1) ^22^. Finally, following hospital discharge for a first episode of AP, a recent study reported 1-year mortality associated with three independent predictors: readmission within 30 days, higher Charlson comorbidity index, and longer hospitalizations ^23^.

### Risk factors

Recent studies address the risk modifiers for AP due to alcohol and gallstones, newly identified risk factors (cannabis), factors responsible for AP in the setting of inflammatory bowel disease (IBD) and end-stage renal disease (ESRD), and the risk of pancreatic cancer after first attack of AP.

When compared with never-smokers, current tobacco use (hazard ratio [HR] 1.75, 95% CI 1.26–2.44) and former tobacco use (HR 1.63, 95% CI 1.18–2.27) are independent risk factors for AP ^24^. The relationship between alcohol exposure and pancreatitis is complex ^25, 26^. In the meta-analysis by Samokhvalov *et al*., the relationship between dose of alcohol and risk of AP was linear (and with no identifiable threshold) in men but non-linear (J-shaped) in women ^27^. Risk of AP in women was decreased with alcohol consumption up to 40 g/day and increased above this amount. The authors speculated that the observed increased risk in women (beyond 40 g/day) was attributed to inclusion of former drinkers in the reference group or possibly the impact of alcohol reducing the risk of biliary pancreatitis ^27^.

The risk of gallstone-related AP may be influenced by diet: increased by consumption of saturated fats, cholesterol, red meat, and eggs ^28^ but decreased by fiber intake ^28^ and, as noted above, alcohol consumption ^29^.

Cannabis is a possible risk factor for toxin-induced AP on the basis of a recent systematic review ^30^. Although the study is limited by having data from only 26 patients, 15 from case reports, eight from a single prospective study, and three from a case series, a clinically meaningful association is likely, particularly between cannabis use and idiopathic pancreatitis, on the basis of follow-up information. Cannabis abstinence completely abolished recurrent attacks of pancreatitis in patients for whom follow-up information was available ^30^.

Clear understanding of the frequency and etiology of AP in the setting of IBD has implications for selecting treatments for this condition. Chen *et al*. searched the National Health Insurance Program administrative database from Taiwan for disease-specific International Classification of Diseases, Ninth Revision, Clinical Modification (ICD-9-CM) billing codes and identified 11,909 patients with IBD and 47,636 age- and sex-matched controls ^31^. After adjusting for comorbidities, they found a 2.93-fold higher HR (95% CI 2.40–3.58) for AP in the IBD versus non-IBD cohorts ^31^. Important limitations of this study are that the diagnosis and etiology of AP are uncertain and are based on administrative ICD-9-CM billing codes without confirming diagnoses by chart review or excluding multiple other causes of AP, including alcohol, medications that may cause AP (for example, azathioprine and mesalamine), familial pancreatitis, and type II autoimmune pancreatitis (AIP) ^32^. Recently, Ramos *et al*. highlighted that gallstones and drug-induced pancreatitis are the most common causes of AP in patients with IBD ^33^. It is important to note that type II AIP has a higher association with IBD ^34^ and that IBD is associated with asymptomatic elevations in serum lipase and amylase ^35^.

Severe renal disease is an established association with pancreatic diseases ^36^. In patients with ESRD, the risk of AP and possibly more severe AP is higher in those who receive peritoneal dialysis compared with hemodialysis ^36– 41^. Recently, Chen *et al*. identified additional independent risk factors for AP in the ESRD population, including older age, being a woman, and having biliary stones or liver disease ^42^.

As recently reviewed ^43^, pancreatic cancer appears to be associated with first-attack pancreatitis with few exceptions ^44^. In a Veterans Affairs National Medical Care Data Set, containing nearly 500,000 patients, 11% of those with diagnosed pancreatic cancer had an attack of AP in the previous 2 years ^45^. The incidence of pancreatic cancer was 1.5% and was greatest within the first year of the attack of AP. The incidence of cancer correlates with age, negligible below age 40 but steadily rising through the fifth to eighth decades. The main message from these studies is to consider and screen for pancreatic cancer as a potential etiology of unexplained AP in patients, particularly those 40 years of age or older. Similar findings were observed in the more recent nationwide matched-cohort study in Denmark, which included 41,669 patients with incident AP who were compared with 208,340 age- and sex-matched controls ^43^. AP was associated with an overall increased absolute risk of pancreatic cancer, which was highest after 2 years of follow-up (0.68%, 95% CI 0.61–0.77%) but persistently increased after 5 years (0.85%, 95% CI 0.76–0.94%) ^43^. The persistent nature of the risk may underscore the importance of inflammation as a cofactor in the development of pancreatic cancer and show that a subset of patients in this Danish cohort may have had underdiagnosed CP.

### Etiology and diagnosis

To date, alcohol and gallstones remain the most prevalent etiologies for AP ^2^. Studies from over 10 years ago reported frequencies of 40–50% and about 20% for biliary AP and alcohol AP, respectively ^22, 46, 47^. A more recent study reported a lower frequency of biliary AP (22%) and a higher frequency of alcohol AP (29%), likely due to inclusion of patients with CP, representing 15% of the cohort of patients with AP. AP due to hypertriglyceridemia, estimated to be about 9% of AP, is less common ^48^.

Guidelines and recent studies of AP raise questions about the threshold above which hypertriglyceridemia causes or poses as an important cofactor for AP. The triglyceride threshold value for causing AP was set as at least 1,000 mg/dL by the ACG and the Endocrine Society and at least 885 mg/dL by the European Society of Cardiology and the European Arteriosclerosis Society ^2, 49, 50^. Pedersen *et al.* provide evidence of a graded risk of AP with hypertriglyceridemia, based on analysis of a prospective cohort of 116,500 individuals with triglyceride measurements from the Copenhagen General Population Study (2003–2015) and the Copenhagen City Heart Study (1976–2003) ^51^. By multivariable analysis, adjusted HRs for AP were much higher with non-fasting mild-to-moderately elevated plasma triglycerides (177–885 mg/dL) compared with normal values (<89 mg/dL) ^51^. Nawaz *et al.* also reported that, in addition to posing a risk for AP, the risk of severe AP (developing POF) increases in proportion to triglyceride value, independent of the underlying cause of AP ^52^. A natural question from a recent systematic review is whether AP is significantly more severe when the cause is hypertriglyceridemia compared with other etiologies, but data are limited ^48^. The same systematic review reported that plasmapheresis effectively decreases circulating triglycerides in patients with AP but has no conclusive mortality benefit ^48^. A significant limitation is that data supporting the efficacy of plasmapheresis are extrapolated primarily from observational studies and case series. The current standard of care is directed toward bowel rest and insulin infusion to reduce triglyceride levels.

Diagnosis of AP is derived from the revised Atlanta classification ^53^. The recommended timing and indications for offering cross-sectional imaging are after 48–72 hours, when a patient experiences no improvement to initial care ^2^. The advantage of magnetic resonance cholangiopancreatography (MRCP) compared with computed tomography (CT) is the capability of identifying choledocholithiasis as small as 3 mm as well as pancreatic duct disruption ^54^. Although specific guideline recommendations advocate more selective use of pancreatic imaging in the early assessment of AP, a recent retrospective study observed no significant decrease in the utilization of early CT or MRCP imaging (within the first 24 hours of care) in the period of 2014–2015 compared with 2006–2007, indicating that quality improvement initiatives are needed to decrease the over-utilization of imaging ^55^.

Clinical applications of endoscopic ultrasound (EUS) and ERCP have evolved for the evaluation of patients with suspected acute biliary pancreatitis. ERCP is not recommended as a pure diagnostic tool, owing to the availability of other diagnostic tests and a complication rate of 5–10% with risks involving PEP, cholangitis, perforation, and hemorrhage ^56^. A recent systematic review of EUS and ERCP in acute biliary pancreatitis included seven studies having a total of 545 patients ^57^. The authors concluded that EUS had lower failure rates and had no complications, and the use of EUS avoided ERCP in 71.2% of cases ^57^.

### Risk stratification

The goals of risk stratification tools in AP are to identify patients at risk for developing major outcomes, including POF, infected pancreatic necrosis, and death. The underlying premise is that predicting the severity of AP within 48 to 72 hours of presentation enables triaging of patients to an appropriate level of care to decrease morbidity and mortality associated with AP. Two recent guidelines affirmed the importance of predicting the severity of AP, using one or more predictive tools ^2, 5^. The recent 2018 AGA technical review does not debate this common-sense approach, including use of dynamic predictive variables over time, but does highlight that there is no published observational study or randomized controlled trial (RCT) investigating whether the use of severity prediction tools impacts clinical outcomes ^3^.

A diverse array of severity prediction tools exists, categorized as clinical scoring systems, single laboratory values, and other variables. Some examples of commonly used clinical scoring systems are Acute Physiology and Chronic Health Evaluation II (APACHE II), BISAP (blood urea nitrogen [BUN], impaired mental status, systemic inflammatory response syndrome, age, and pleural effusion), early warning system (EWS), Glasgow-Imrie score, and Japanese severity score. BISAP is a validated clinical scoring system that predicts in-hospital mortality on the basis of five variables: BUN of more than 25 mg/dL, impaired mental status, systemic inflammatory response syndrome, age of more than 60, or presence of pleural effusion ^58^. In 2010, Papachristou *et al*. determined that BISAP scoring is just as accurate as APACHE II, CT severity index, and Ranson’s in predicting the severity and prognosis of AP ^59^. A subsequent comparative analysis by the same investigators concluded that available predictive tools have limited clinical utility by having only moderate predictive value for POF and mortality ^60^. More elaborate and automated machine learning algorithms have also been developed for predicting severity, designed using artificial neural networks and metabonomics technology, but the incorporation of these algorithms into clinical applications remains challenging ^61– 63^.

## Recent advances in early treatment of acute pancreatitis

### Literature review and definitions

The AP literature contains heterogeneous definitions of severe AP and of what constitutes a major outcome in AP. This limitation, particularly in older literature, served as the impetus for the 2012 revision of the Atlanta Criteria for AP ^53^. To extract from the literature meaningful summary evidence and estimates for the AGA Clinical Guideline on early medical management of AP, the 2018 AGA technical review applied precise definitions to each step of the review process, and the emphasis was on clear definitions of primary outcomes of clinical importance in AP, including death, persistent single organ failure (PSOF), or persistent multiple organ failure (PMOF), each requiring a duration of more than 48 hours, and infected pancreatic or peri-pancreatic necrosis or both ^3, 4^. For these reasons, our review of early treatment of AP preferentially weighs recommendations in favor of those espoused by the 2018 AGA technical review and clinical guideline.

### Intravenous fluid administration

Supportive care with the use of intravenous fluid hydration is a mainstay of treatment for AP in the first 12–24 hours. Hypovolemia in AP is driven by third spacing and intravascular volume depletion ^64^. Guidelines advocate for early fluid resuscitation to correct intravascular depletion in order to reduce morbidity and mortality associated with AP ^2, 3, 5^. However, as Haydock *et al.* point out, there is a deficiency of high-quality data to establish firm recommendations ^65^. The 2018 AGA guidelines endorse a conditional recommendation for using goal-directed therapy for initial fluid management ^4^, do not recommend for or against normal saline versus lactated Ringer’s (LR), but do advise against the use of hydroxyethyl starch fluids ^4^. The AGA guidelines and the technical review acknowledge that evidence was weak in support of goal-directed therapy ^3, 4^, which was a pre-defined study arm in four of seven RCTs reviewed and had no significant reduction in PMOF, mortality, or pancreatic necrosis compared with usual care. As the authors noted, interpretation of the data was limited by the absence of other critical outcomes in these trials (infected pancreatic necrosis), lack of uniformity of specific outcomes and definitions of transient and POF, few trials, and risk of bias. As illustrated in a recent review and RCT, potential endpoints of goal-directed therapy over the first 12–24 hours may include reducing serum BUN and hematocrit at various intervals after initial fluid administration ^66, 67^. Recent hypothesis-generating efforts by DiMagno *et al*. illustrate how integrating a goal-directed fluid therapy algorithm with a web-based protocol and paging alert system shows promise for decreasing variability in care and shortening hospital LOS ^68^.

### Feeding

The focus of nutrition in the management of AP has undergone several paradigm shifts. Prior recommendations, for patients to remain *nil per os*, were aimed at decreasing stimulation of exocrine pancreatic secretion, which in theory would decrease enzyme-driven inflammation and promote earlier recovery. More recently, the focus has shifted toward early initiation of enteral feeding to protect the gut-mucosal barrier. Current guidelines advocate for early oral feeding (within 24 hours) in mild AP ^3, 4^. In the AGA technical review of 11 RCTs of early versus delayed feeding in AP, there was no significant impact on outcomes of early- versus delayed oral feeding, with only non-significant trends towards lower rates of mortality and persistent single organ failure ^3^. These negative findings mirror observations of the landmark Dutch PYTHON trial entitled “Early versus on-demand naso-enteric tube feeding in acute pancreatitis ^69^. In subset analyses, remaining NPO when compared with early oral feeding, had a 2.5-fold higher risk for interventions for necrosis and no significant impact on other outcome measures (1 trial) ^3^. Because timing of feeding varied across studies, there is no clear cutoff point for initiating feeding for those with severe AP; a practical approach is to initiate feeding within 24–72 hours and offer enteral nutrition (EN) for those intolerant to oral feeds. In severe AP and moderately severe AP (for example, necrotizing pancreatitis), EN is recommended over parenteral nutrition (PN) ^3, 4^. In the AGA technical review of 12 RCTs of EN versus PN in AP, there was clear evidence that EN reduced the risk of infected peri-pancreatic necrosis (OR 0.28, 95% CI 0.15–0.51), single organ failure (OR 0.25, 95% CI 0.10–0.62), and MOF (OR 0.41, 95% CI 0.27–0.63) ^3^. Finally, the AGA guidelines provide a conditional recommendation on providing EN support through either the nasogastric or the nasoenteral route ^4^. Data from the supporting AGA technical review were considered low-quality evidence, limited to three RCTs having significant methodologic limitations (for example, using different definitions of severe AP) and having a high risk of detection bias due to issues with outcome assessment ^3^. Overall, EN is favored over PN for nutritional support in severe AP, but further studies are required to determine the optimal timing, rate, and formulation of EN in severe AP.

### Antibiotics

Recent guidelines do not support the use of prophylactic antibiotics to prevent infection in necrotizing AP and severe AP ^2, 4, 5^. The recent AGA technical review reaches similar conclusions. In an analysis of 10 RCTs involving 701 patients, limited to the higher-quality RCTs published after 2002, prophylactic antibiotics did not reduce infected pancreatic or peri-pancreatic necrosis, PSOF, or mortality ^3^.

### Probiotics

Recent guidelines advocate against the use of probiotics for severe AP ^2^. The largest double-blinded RCT, published by Besselink *et al*., demonstrated that probiotic prophylaxis did not reduce the risk of infectious complications and was associated with a higher incidence of bowel ischemia (9/153 versus 0/145, *p* = 0.004) and greater mortality (risk ratio [RR] = 2.53, 95% CI = 1.22–5.25) ^70^. In a recent systematic review and meta-analysis of six heterogeneous RCTs, Gou *et al*. reported that probiotics did not reduce pancreatic infection rates, hospital LOS, or mortality ^71^.

### Timing of endoscopic retrograde cholangiopancreatography in acute biliary pancreatitis

There is universal agreement for offering urgent ERCP (within 24 hours) in biliary AP complicated by cholangitis ^2, 4, 5, 72^. In the absence of cholangitis, the timing of ERCP for AP with persistent biliary obstruction is less clear ^2, 4, 5^. Prior guidelines, the Cochrane systematic review, and the recent 2018 AGA clinical guidelines and technical review argue against routine use of urgent ERCP for acute biliary pancreatitis ^2– 5, 72^. The AGA technical review of eight RCTs with 935 patients concludes that urgent ERCP compared with conservative management in acute biliary pancreatitis had no significant impact on major outcomes (mortality, organ failure, infected pancreatic necrosis, and total necrotizing pancreatitis) ^3^. Findings were similar in the subgroup analysis of studies that clearly excluded patients with biliary obstruction. Data were sparse and from a single RCT, suggesting that urgent ERCP for persistent biliary obstruction significantly impacted secondary outcomes (as classified in the AGA technical review); urgent ERCP compared with conservative treatment significantly shortened hospital LOS (9.5 days versus 17.0 days, 95% CI = −12.64 to −4.96) ^3, 73^. Based on the significant limitations across trials, including significant heterogeneity and exclusion of patients with cholangitis in recent trials, the AGA technical review considered the overall quality of data low and offered a conditional recommendation against urgent ERCP in patients with biliary AP and no cholangitis ^3^.

There are limited data to guide decision making of when *non-urgent* ERCP should be performed in hospitalized patients with acute biliary pancreatitis with persistent obstruction and no cholangitis. The Cochrane analysis concluded that ERCP should be performed within 72 hours, which associates with a non-significant trend toward reduction in local and systemic complications related to AP ^72^. In support of this recommendation, Lee *et al*. recently performed a retrospective comparison of outcomes in 73 patients with acute biliary pancreatitis with biliary obstruction without cholangitis treated with either *urgent* ERCP (<24 hours) or *early* ERCP (24–72 hours) ^74^. Overall, timing of ERCP had no impact on hospital LOS (5.9 versus 5.7 days, *p* value = 0.174), post-ERCP complications (15% versus 2.6%, *p* value = 0.113), and complications due to pancreatitis (2.6% versus 5.8%), regardless of severity ^74^. Significant limitations of the study (retrospective design and small sample size) underscore the need for an appropriately sized RCT to determine whether urgent versus early ERCP differentially impacts outcomes for persistent biliary obstruction in AP. Practical and informative recommendations for designing future studies were offered in the AGA technical review: “The timing of the ERCP intervention should be 24–48 hours after diagnosis (24 hours to allow spontaneous passage of stone and 48 hours to ensure that prolonged biliary obstruction does not occur)” ^3^.

### Alcohol and smoking cessation

Drinking alcohol and smoking are known independent risk factors for recurrent episodes of AP and CP ^75– 77^. Recently published guidelines from the AGA advocate for brief alcohol intervention during hospitalization for alcohol-induced AP ^4^. To date, only one RCT that addresses the impact of alcohol counseling on recurrent bouts of AP has been identified ^78^. Patients with first-attack AP associated with alcohol were randomly assigned to a single counseling session at the initial hospitalization versus counseling every 6 months for 2 years. The intention-to-treat analysis showed significantly fewer recurrent episodes of AP in the repeated versus single intervention groups (5/59 versus 13/61, *p* value = 0.042) ^78^. There was no significant difference in recurrent AP or hospital readmission rates after excluding patients who dropped out, died, or were identified as having previous episodes of pancreatitis or other etiologies of pancreatitis, a negative finding likely attributable in part to small sample size. As further support for common-sense recommendations to abstain from alcohol, the authors provided follow-up data for 18 subjects remaining abstinent over a mean of 5.15 years (range of 1.83–9.13 years) and reported that abstainers did not have a repeat episode of pancreatitis ^79^.

Cessation of smoking, an established independent risk factor of AP and CP, should also be recommended as part of the management of AP. To determine the impact of smoking cessation on prevention of first-attack AP, Sadr-Azodi *et al*. prospectively studied 84,667 Swedish men and women over a median of 12 years of follow-up and compared 307 patients who had non-gallstone AP with a comparable control group without AP ^80^. The authors reported that the frequency of non-gallstone AP was double in current smokers (≥20 pack-years) compared with never-smokers (RR = 2.29, 95% CI 1.63 to 3.22, *p* <0.01) and that the risk of non-gallstone AP fell to the baseline risk of never-smokers after 20 years of smoking cessation ^80^.

### Cholecystectomy

Evidence supports same-admission cholecystectomy for mild gallstone AP, a recommendation of published guidelines including the 2018 AGA clinical guideline ^2, 4, 5^. In the technical review accompanying the AGA guideline, Vege *et al*. ^3^ identified one RCT that evaluated the effect of same-hospitalization versus delayed (post-discharge) cholecystectomy on outcomes in patients with mild acute gallstone pancreatitis ^81^. Compared with delayed cholecystectomy, same-admission cholecystectomy significantly reduced gallstone-related complications (OR 0.24, 95% CI 0.09–0.61) ^81^ and readmissions for recurrent pancreatitis and pancreaticobiliary complications ^81^ without having a significant impact on mortality during a 6-month follow-up period ^81^. As discussed in the AGA technical review, it remains controversial whether timing of surgery during the index hospitalization has a meaningful impact on outcomes ^3^. Delaying cholecystectomy for 6 weeks in patients with moderate to severe gallstone AP appears to reduce morbidity, including the development of infected collections, and mortality ^3^. In patients unfit for surgery, offering ERCP with biliary sphincterotomy appears to reduce the risk of recurrent acute biliary pancreatitis but not other biliary complications ^82– 84^.

### Chemoprevention and intravenous fluid management of post-endoscopic retrograde cholangiopancreatography pancreatitis

A systematic review of data from worldwide RCTs suggests that the incidence of PEP increased from 7.7% to 10% in the periods before and after 2000 ^85^, respectively, primarily because of an increase in mild PEP from 2.9% to 5.9%. An explanation is likely complex but may be, in part, the increasing use of ERCP as a therapeutic procedure and decreasing use as a diagnostic procedure. With broader adoption of effective preventive therapies for PEP, there is promise that this trend may change.

Accumulating data support the effectiveness of chemoprevention and fluid administration to prevent PEP. Multiple RCTs, meta-analyses, and systematic reviews indicate that rectal non-steroidal anti-inflammatory drugs (NSAIDs) (diclofenac or indomethacin) reduce PEP onset ^86– 89^ and moderate to severe PEP. The literature is inconclusive regarding whether rectal NSAIDs should be administered prior to or immediately after ERCP ^90, 91^. Oral NSAIDs do not prevent PEP ^92– 94^. In 2014, two hypothesis-generating retrospective studies reported that greater peri-procedural intravenous fluid volume was an independent protective factor against moderate to severe PEP ^95^ and was associated with shorter hospital LOS ^96^. Very recent meta-analyses and RCTs support using LR prior to ERCP to prevent PEP ^97– 100^ and to reduce moderate to severe PEP ^97, 98^. Interestingly, a recent RCT shows that the combination of rectal indomethacin and LR compared with combination placebo and normal saline reduced the risk of PEP in high-risk patients ^101^. An ongoing multicenter Dutch “FLUYT” RCT aims to determine the optimal combination of rectal NSAIDs and peri-procedural infusion of intravenous fluids to reduce the incidence of PEP and moderate to severe PEP ^102^. In particular, this study will yield practical information regarding the type and rate of fluids to administer to patients: no fluids versus normal saline (1.5 mL/kg per hour) versus LR (20 mL/kg rapid infusion over 60 minutes and then 3 mL/kg per hour).

Chemoprevention of PEP using a variety of other agents has not been fruitful. The most recent meta-analyses and RCTs have demonstrated that allopurinol ^103^, corticosteroids ^104– 106^, somatostatin and their analogues ^107^, heparin ^108^, and nitroglycerin ^87, 109^ have an inconclusive or non-significant effect on reducing the risk of PEP. Whereas the protease inhibitor nafamostat reduces the risk of PEP ^87, 110^, it does not reduce the risk of PEP in high-risk patients, has a high cost, and requires a prolonged 7- to 25-hour infusion that diminishes the practicality of offering this to patients ^110^.

### Implications for clinical practice

The diagnosis and optimal management of AP require a systematic approach with multidisciplinary decision making. Morbidity and mortality in AP are driven by early or late POF, and the latter often is triggered by infected necrosis. Risk stratification of these patients at the point of contact is a common-sense approach to enable triaging of patients to the appropriate level of care.

Regardless of pancreatitis severity, recommended treatment interventions include goal-directed intravenous fluid resuscitation, early feeding by mouth or by enteral tube when necessary, avoidance of prophylactic antibiotics, avoidance of probiotics, and urgent ERCP for patients with acute biliary pancreatitis complicated by cholangitis. Management decisions not discussed in this review include critical care management in AP (for example, abdominal compartment syndrome ^6^) and late decision making for pancreatitis complicated by infected necrosis, which involves source control with antibiotics and step-up therapy using a combination of percutaneous drainage, endoscopic management, and surgical intervention ^7– 10^. Key measures for preventing hospital readmission and pancreatitis include same-admission cholecystectomy for acute biliary pancreatitis and alcohol and smoking cessation. Preventive measures for PEP in patients undergoing ERCP include rectal indomethacin and peri-procedural fluid resuscitation. Table 1 summarizes recent major advances in AP that may affect clinical practice.

**Table 1.** Recent advances in epidemiology, evaluation, and management of acute pancreatitis.

1. Incidence of acute pancreatitis (AP) is increasing, but mortality is decreasing
2. Alcohol and gallstones remain the most common causes of AP
3. Smoking is an independent risk factor for pancreatitis
4. Cannabis is a possible risk factor for toxin-induced AP
5. In inflammatory bowel disease, AP is typically due to gallstones or medications
6. In severe renal disease, risk of AP is higher with ongoing peritoneal dialysis
7. Pancreatic cancer is an uncommon but established cause of first-attack pancreatitis
8. The risk of AP and severe AP appears to increase in proportion to triglyceride value
9. Cross-sectional imaging remains over-utilized during the initial evaluation of AP
10. Risk stratification tools have moderate predictive value for severe AP
11. Goal-directed fluid therapy (FT) is recommended as early treatment of AP
12. Recommended fluids for FT are normal saline or lactated Ringer’s, not hydroxyethyl starch
13. Initiation of early oral feeding is recommended, beginning within 24 hours, for mild AP
14. Enteral nutritional support is favored over parental nutrition in severe AP
15. Prophylactic antibiotics are not recommended for necrotizing pancreatitis
16. Probiotics are not recommended for severe AP
17. Urgent endoscopic retrograde cholangiopancreatography (ERCP) (<24 hours) is recommended for acute biliary pancreatitis complicated by cholangitis
18. Routine use of urgent ERCP is not recommended for acute biliary pancreatitis
19. Same-hospitalization and repeated alcohol cessation counseling is recommended for alcohol-induced AP
20. Same-admission cholecystectomy is recommended for mild acute biliary pancreatitis
21. Rectal indomethacin and peri-procedural FT each reduce post-ERCP pancreatitis; combination therapy requires study

## Abbreviations

ACG, American College of Gastroenterology; AGA, American Gastroenterological Association; AIP, autoimmune pancreatitis; AP, acute pancreatitis; APACHE II, Acute Physiology and Chronic Health Evaluation II; BISAP, blood urea nitrogen, impaired mental status, systemic inflammatory response syndrome, age, and pleural effusion; BUN, blood urea nitrogen; CI, confidence interval; CP, chronic pancreatitis; CT, computed tomography; EN, enteral nutrition; ERCP, endoscopic retrograde cholangiopancreatography; ESRD, end-stage renal disease; EUS, endoscopic ultrasound; HR, hazard ratio; IBD, inflammatory bowel disease; ICD-9, International Classification of Diseases, Ninth Revision; LOS, length of stay; LR, lactated Ringer’s; MOF, multiple organ failure; MRCP, magnetic resonance cholangiopancreatography; NIS, nationwide inpatient sample; NSAID, non-steroidal anti-inflammatory drug; OR, odds ratio; PEP, post-endoscopic retrograde cholangiopancreatography pancreatitis; PMOF, persistent multiple organ failure; PN, parenteral nutrition; POF, persistent organ failure; PSOF, persistent single organ failure; RCT, randomized controlled trial; RR, risk ratio.

## References

[1] Peery AF Dellon ES Lund J: Burden of gastrointestinal disease in the United States: 2012 update. *Gastroenterology.* 2012;143(5):1179–87.e1-3. 10.1053/j.gastro.2012.08.002 22885331 PMC3480553

[2] Tenner S Baillie J DeWitt J: American College of Gastroenterology guideline: management of acute pancreatitis. *Am J Gastroenterol.* 2013;108(9):1400–15; 1416. 10.1038/ajg.2013.218 23896955

[3] Vege SS DiMagno MJ Forsmark CE: Initial Medical Treatment of Acute Pancreatitis: American Gastroenterological Association Institute Technical Review. *Gastroenterology.* 2018;154(4):1103–39. 10.1053/j.gastro.2018.01.031 29421596

[4] Crockett SD Wani S Gardner TB: American Gastroenterological Association Institute Guideline on Initial Management of Acute Pancreatitis. *Gastroenterology.* 2018;154(4):1096–101. 10.1053/j.gastro.2018.01.032 29409760

[5] Working Group IAP/APA Acute Pancreatitis Guidelines: IAP/APA evidence-based guidelines for the management of acute pancreatitis. *Pancreatology.* 2013;13(4 Suppl 2):e1–15. 10.1016/j.pan.2013.07.063 24054878

[6] van Brunschot S Schut AJ Bouwense SA: Abdominal compartment syndrome in acute pancreatitis: a systematic review. *Pancreas.* 2014;43(5):665–74. 10.1097/MPA.0000000000000108 24921201

[7] Bakker OJ van Santvoort HC van Brunschot S: Endoscopic transgastric vs surgical necrosectomy for infected necrotizing pancreatitis: a randomized trial. *JAMA.* 2012;307(10):1053–61. 10.1001/jama.2012.276 22416101

[8] Freeman ML Werner J van Santvoort HC: Interventions for necrotizing pancreatitis: summary of a multidisciplinary consensus conference. *Pancreas.* 2012;41(8):1176–94. 10.1097/MPA.0b013e318269c660 23086243

[9] Mouli VP Sreenivas V Garg PK: Efficacy of conservative treatment, without necrosectomy, for infected pancreatic necrosis: a systematic review and meta-analysis. *Gastroenterology.* 2013;144(2):333–340.e2. 10.1053/j.gastro.2012.10.004 23063972

[10] van Santvoort HC Besselink MG Bakker OJ: A step-up approach or open necrosectomy for necrotizing pancreatitis. *N Engl J Med.* 2010;362(16):1491–502. 10.1056/NEJMoa0908821 20410514

[11] Sellers ZM MacIsaac D Yu H: Nationwide Trends in Acute and Chronic Pancreatitis Among Privately Insured Children and Non-Elderly Adults in the United States, 2007-2014. *Gastroenterology.* 2018;155(2):469–478.e1. 10.1053/j.gastro.2018.04.013 29660323 PMC6067969

[12] Krishna SG Kamboj AK Hart PA: The Changing Epidemiology of Acute Pancreatitis Hospitalizations: A Decade of Trends and the Impact of Chronic Pancreatitis. *Pancreas.* 2017;46(4):482–8. 10.1097/MPA.0000000000000783 28196021 PMC5435121

[13] Brown A Young B Morton J: Are health related outcomes in acute pancreatitis improving? An analysis of national trends in the U.S. from 1997 to 2003. *JOP.* 2008;9(4):408–14. 18648131

[14] Fagenholz PJ Castillo CF Harris NS: Increasing United States hospital admissions for acute pancreatitis, 1988-2003. *Ann Epidemiol.* 2007;17(7):491–7. 10.1016/j.annepidem.2007.02.002 17448682

[15] McNabb-Baltar J Ravi P Isabwe GA: A population-based assessment of the burden of acute pancreatitis in the United States. *Pancreas.* 2014;43(5):687–91. 10.1097/MPA.0000000000000123 24694835

[16] Agarwal S George J Padhan RK: Reduction in mortality in severe acute pancreatitis: A time trend analysis over 16 years. *Pancreatology.* 2016;16(2):194–9. 10.1016/j.pan.2016.01.012 26915280

[17] Andersen AM Novovic S Ersbøll AK: Mortality in alcohol and biliary acute pancreatitis. *Pancreas.* 2008;36(4):432–4. 10.1097/MPA.0b013e31815ceae5 18437092

[18] Goyal H Smith B Bayer C: Differences in Severity and Outcomes Between Hypertriglyceridemia and Alcohol-Induced Pancreatitis. *N Am J Med Sci.* 2016;8(2):82–7. 10.4103/1947-2714.177307 27042605 PMC4791903

[19] Huh JH Jeon H Park SM: Diabetes Mellitus is Associated With Mortality in Acute Pancreatitis. *J Clin Gastroenterol.* 2018;52(2):178–83. 28009683 10.1097/MCG.0000000000000783

[20] Wu BU Johannes RS Kurtz S: The impact of hospital-acquired infection on outcome in acute pancreatitis. *Gastroenterology.* 2008;135(3):816–20. 10.1053/j.gastro.2008.05.053 18616944 PMC2570951

[21] Gardner TB Vege SS Pearson RK: Fluid resuscitation in acute pancreatitis. *Clin Gastroenterol Hepatol.* 2008;6(10):1070–6. 10.1016/j.cgh.2008.05.005 18619920

[22] Krishna SG Hinton A Oza V: Morbid Obesity Is Associated With Adverse Clinical Outcomes in Acute Pancreatitis: A Propensity-Matched Study. *Am J Gastroenterol.* 2015;110(11):1608–19. 10.1038/ajg.2015.343 26482857

[23] Lee PJ Bhatt A Lopez R: Thirty-Day Readmission Predicts 1-Year Mortality in Acute Pancreatitis. *Pancreas.* 2016;45(4):561–4. 10.1097/MPA.0000000000000463 26390423

[24] Majumder S Gierisch JM Bastian LA: The association of smoking and acute pancreatitis: a systematic review and meta-analysis. *Pancreas.* 2015;44(4):540–6. 10.1097/MPA.0000000000000301 25872130

[25] DiMagno MJ: Oktoberfest binge drinking and acute pancreatitis: is there really no relationship? *Clin Gastroenterol Hepatol.* 2011;9(11):920–2. 10.1016/j.cgh.2011.07.022 21819953

[26] Phillip V Huber W Hagemes F: Incidence of acute pancreatitis does not increase during Oktoberfest, but is higher than previously described in Germany. *Clin Gastroenterol Hepatol.* 2011;9(11):995–1000.e3. 10.1016/j.cgh.2011.06.016 21723238

[27] Samokhvalov AV Rehm J Roerecke M: Alcohol Consumption as a Risk Factor for Acute and Chronic Pancreatitis: A Systematic Review and a Series of Meta-analyses. *EBioMedicine.* 2015;2(12):1996–2002. 10.1016/j.ebiom.2015.11.023 26844279 PMC4703772

[28] Setiawan VW Pandol SJ Porcel J: Dietary Factors Reduce Risk of Acute Pancreatitis in a Large Multiethnic Cohort. *Clin Gastroenterol Hepatol.* 2017;15(2):257–265.e3. 10.1016/j.cgh.2016.08.038 27609706 PMC5241169

[29] Morton C Klatsky AL Udaltsova N: Smoking, coffee, and pancreatitis. *Am J Gastroenterol.* 2004;99(4):731–8. 10.1111/j.1572-0241.2004.04143.x 15089909

[30] Barkin JA Nemeth Z Saluja AK: Cannabis-Induced Acute Pancreatitis: A Systematic Review. *Pancreas.* 2017;46(8):1035–8. 10.1097/MPA.0000000000000873 28796137

[31] Chen YT Su JS Tseng CW: Inflammatory bowel disease on the risk of acute pancreatitis: A population-based cohort study. *J Gastroenterol Hepatol.* 2016;31(4):782–7. 10.1111/jgh.13171 26412125

[32] Bermejo F Lopez-Sanroman A Taxonera C: Acute pancreatitis in inflammatory bowel disease, with special reference to azathioprine-induced pancreatitis. *Aliment Pharmacol Ther.* 2008;28(5):623–8. 10.1111/j.1365-2036.2008.03746.x 18513380

[33] Ramos LR Sachar DB DiMaio CJ: Inflammatory Bowel Disease and Pancreatitis: A Review. *J Crohns Colitis.* 2016;10(1):95–104. 10.1093/ecco-jcc/jjv153 26351384

[34] Lorenzo D Maire F Stefanescu C: Features of Autoimmune Pancreatitis Associated With Inflammatory Bowel Diseases. *Clin Gastroenterol Hepatol.* 2018;16(1):59–67. 10.1016/j.cgh.2017.07.033 28782667

[35] Heikius B Niemelä S Lehtola J: Elevated pancreatic enzymes in inflammatory bowel disease are associated with extensive disease. *Am J Gastroenterol.* 1999;94(4):1062–9. 10.1111/j.1572-0241.1999.01015.x 10201484

[36] Avram MM: High prevalence of pancreatic disease in chronic renal failure. *Nephron.* 1977;18(1):68–71. 10.1159/000180768 846627

[37] Lankisch PG Weber-Dany B Maisonneuve P: Frequency and severity of acute pancreatitis in chronic dialysis patients. *Nephrol Dial Transplant.* 2008;23(4):1401–5. 10.1093/ndt/gfm769 18065830

[38] Owyang C Miller LJ DiMagno EP: Gastrointestinal hormone profile in renal insufficiency. *Mayo Clin Proc.* 1979;54(12):769–73. 513844

[39] Owyang C Miller LJ DiMagno EP: Pancreatic exocrine function in severe human chronic renal failure. *Gut.* 1982;23(5):357–61. 10.1136/gut.23.5.357 6804312 PMC1419670

[40] Quraishi ER Goel S Gupta M: Acute pancreatitis in patients on chronic peritoneal dialysis: an increased risk? *Am J Gastroenterol.* 2005;100(10):2288–93. 10.1111/j.1572-0241.2005.41646.x 16181382

[41] Vaziri ND Dure-Smith B Miller R: Pancreatic pathology in chronic dialysis patients--an autopsy study of 78 cases. *Nephron.* 1987;46(4):347–9. 10.1159/000184387 3658062

[42] Chen HJ Wang JJ Tsay WI: Epidemiology and outcome of acute pancreatitis in end-stage renal disease dialysis patients: a 10-year national cohort study. *Nephrol Dial Transplant.* 2017;32(10):1731–6. 10.1093/ndt/gfw400 28088773

[43] Kirkegård J Cronin-Fenton D Heide-Jørgensen U: Acute Pancreatitis and Pancreatic Cancer Risk: A Nationwide Matched-Cohort Study in Denmark. *Gastroenterology.* 2018;154(6):1729–36. 10.1053/j.gastro.2018.02.011 29432727

[44] Karlson BM Ekbom A Josefsson S: The risk of pancreatic cancer following pancreatitis: an association due to confounding? *Gastroenterology.* 1997;113(2):587–92. 10.1053/gast.1997.v113.pm9247480 9247480

[45] Munigala S Kanwal F Xian H: Increased risk of pancreatic adenocarcinoma after acute pancreatitis. *Clin Gastroenterol Hepatol.* 2014;12(7):1143–1150.e1. 10.1016/j.cgh.2013.12.033 24440214

[46] Frey CF Zhou H Harvey DJ: The incidence and case-fatality rates of acute biliary, alcoholic, and idiopathic pancreatitis in California, 1994–2001. *Pancreas.* 2006;33(4):336–44. 10.1097/01.mpa.0000236727.16370.99 17079936

[47] Yadav D Lowenfels AB: Trends in the epidemiology of the first attack of acute pancreatitis: a systematic review. *Pancreas.* 2006;33(4):323–30. 10.1097/01.mpa.0000236733.31617.52 17079934

[48] Carr RA Rejowski BJ Cote GA: Systematic review of hypertriglyceridemia-induced acute pancreatitis: A more virulent etiology? *Pancreatology.* 2016;16(4):469–76. 10.1016/j.pan.2016.02.011 27012480

[49] Berglund L Brunzell JD Goldberg AC: Evaluation and treatment of hypertriglyceridemia: an Endocrine Society clinical practice guideline. *J Clin Endocrinol Metab.* 2012;97(9):2969–89. 10.1210/jc.2011-3213 22962670 PMC3431581

[50] Catapano AL Reiner Z De Backer G: ESC/EAS Guidelines for the management of dyslipidaemias The Task Force for the management of dyslipidaemias of the European Society of Cardiology (ESC) and the European Atherosclerosis Society (EAS). *Atherosclerosis.* 2011;217(1):3–46. 10.1016/j.atherosclerosis.2011.06.028 21882396

[51] Pedersen SB Langsted A Nordestgaard BG: Nonfasting Mild-to-Moderate Hypertriglyceridemia and Risk of Acute Pancreatitis. *JAMA Intern Med.* 2016;176(12):1834–42. 10.1001/jamainternmed.2016.6875 27820614

[52] Nawaz H Koutroumpakis E Easler J: Elevated serum triglycerides are independently associated with persistent organ failure in acute pancreatitis. *Am J Gastroenterol.* 2015;110(10):1497–503. 10.1038/ajg.2015.261 26323188

[53] Banks PA Bollen TL Dervenis C: Classification of acute pancreatitis--2012: revision of the Atlanta classification and definitions by international consensus. *Gut.* 2013;62(1):102–11. 10.1136/gutjnl-2012-302779 23100216

[54] Stimac D Miletić D Radić M: The role of nonenhanced magnetic resonance imaging in the early assessment of acute pancreatitis. *Am J Gastroenterol.* 2007;102(5):997–1004. 10.1111/j.1572-0241.2007.01164.x 17378903

[55] Jin DX McNabb-Baltar JY Suleiman SL: Early Abdominal Imaging Remains Over-Utilized in Acute Pancreatitis. *Dig Dis Sci.* 2017;62(10):2894–9. 10.1007/s10620-017-4720-x 28840381

[56] Freeman ML: Complications of endoscopic retrograde cholangiopancreatography: avoidance and management. *Gastrointest Endosc Clin N Am.* 2012;22(3):567–86. 10.1016/j.giec.2012.05.001 22748249

[57] De Lisi S Leandro G Buscarini E: Endoscopic ultrasonography versus endoscopic retrograde cholangiopancreatography in acute biliary pancreatitis: a systematic review. *Eur J Gastroenterol Hepatol.* 2011;23(5):367–74. 10.1097/MEG.0b013e3283460129 21487299

[58] Wu BU Johannes RS Sun X: The early prediction of mortality in acute pancreatitis: a large population-based study. *Gut.* 2008;57(12):1698–703. 10.1136/gut.2008.152702 18519429

[59] Papachristou GI Muddana V Yadav D: Comparison of BISAP, Ranson's, APACHE-II, and CTSI scores in predicting organ failure, complications, and mortality in acute pancreatitis. *Am J Gastroenterol.* 2010;105(2):435–41; quiz 442. 10.1038/ajg.2009.622 19861954

[60] Mounzer R Langmead CJ Wu BU: Comparison of existing clinical scoring systems to predict persistent organ failure in patients with acute pancreatitis. *Gastroenterology.* 2012;142(7):1476–82; quiz e15-6. 10.1053/j.gastro.2012.03.005 22425589

[61] Pearce CB Gunn SR Ahmed A: Machine learning can improve prediction of severity in acute pancreatitis using admission values of APACHE II score and C-reactive protein. *Pancreatology.* 2006;6(1–2):123–31. 10.1159/000090032 16327290

[62] van den Heever M Mittal A Haydock M: The use of intelligent database systems in acute pancreatitis--a systematic review. *Pancreatology.* 2014;14(1):9–16. 10.1016/j.pan.2013.11.010 24555973

[63] Xu H Zhang L Kang H: Serum Metabonomics of Mild Acute Pancreatitis. *J Clin Lab Anal.* 2016;30(6):990–8. 10.1002/jcla.21969 27169745 PMC6807221

[64] Forsmark ChE Vege SS Wilcox CM: Acute Pancreatitis. *N Engl J Med.* 2017;376(6):598–9. 10.1056/NEJMc1616177 28177868

[65] Haydock MD Mittal A Wilms HR: Fluid therapy in acute pancreatitis: anybody's guess. *Ann Surg.* 2013;257(2):182–8. 10.1097/SLA.0b013e31827773ff 23207241

[66] Buxbaum JL Quezada M Da B: Early Aggressive Hydration Hastens Clinical Improvement in Mild Acute Pancreatitis. *Am J Gastroenterol.* 2017;112(5):797–803. 10.1038/ajg.2017.40 28266591

[67] DiMagno MJ: Clinical update on fluid therapy and nutritional support in acute pancreatitis. *Pancreatology.* 2015;15(6):583–8. 10.1016/j.pan.2015.09.005 26454418

[68] DiMagno MJ Wamsteker EJ Rizk RS: A combined paging alert and web-based instrument alters clinician behavior and shortens hospital length of stay in acute pancreatitis. *Am J Gastroenterol.* 2014;109(3):306–15. 10.1038/ajg.2013.282 24594946 PMC5565843

[69] Bakker OJ van Brunschot S van Santvoort HC: Early versus on-demand nasoenteric tube feeding in acute pancreatitis. *N Engl J Med.* 2014;371(21):1983–93. 10.1056/NEJMoa1404393 25409371

[70] Besselink MG van Santvoort HC Buskens E: Probiotic prophylaxis in predicted severe acute pancreatitis: a randomised, double-blind, placebo-controlled trial. *Lancet.* 2008;371(9613):651–9. 10.1016/S0140-6736(08)60207-X 18279948

[71] Gou S Yang Z Liu T: Use of probiotics in the treatment of severe acute pancreatitis: a systematic review and meta-analysis of randomized controlled trials. *Crit Care.* 2014;18(2):R57. 10.1186/cc13809 24684832 PMC4056604

[72] Tse F Yuan Y: Early routine endoscopic retrograde cholangiopancreatography strategy versus early conservative management strategy in acute gallstone pancreatitis. *Cochrane Database Syst Rev.* 2012; (5):CD009779. 10.1002/14651858.CD009779.pub2 22592743 PMC11491195

[73] Neoptolemos JP Carr-Locke DL London NJ: Controlled trial of urgent endoscopic retrograde cholangiopancreatography and endoscopic sphincterotomy versus conservative treatment for acute pancreatitis due to gallstones. *Lancet.* 1988;2(8678):979–83. 10.1016/S0140-6736(88)90740-4 2902491

[74] Lee HS Chung MJ Park JY: Urgent endoscopic retrograde cholangiopancreatography is not superior to early ERCP in acute biliary pancreatitis with biliary obstruction without cholangitis. *PLoS One.* 2018;13(2):e0190835. 10.1371/journal.pone.0190835 29401491 PMC5798765

[75] Ahmed Ali U Issa Y Hagenaars JC: Risk of Recurrent Pancreatitis and Progression to Chronic Pancreatitis After a First Episode of Acute Pancreatitis. *Clin Gastroenterol Hepatol.* 2016;14(5):738–46. 10.1016/j.cgh.2015.12.040 26772149

[76] Sankaran SJ Xiao AY Wu LM: Frequency of progression from acute to chronic pancreatitis and risk factors: a meta-analysis. *Gastroenterology.* 2015;149(6):1490–1500.e1. 10.1053/j.gastro.2015.07.066 26299411

[77] Ye X Lu G Huai J: Impact of smoking on the risk of pancreatitis: a systematic review and meta-analysis. *PLoS One.* 2015;10(4):e0124075. 10.1371/journal.pone.0124075 25879541 PMC4399880

[78] Nordback I Pelli H Lappalainen-Lehto R: The recurrence of acute alcohol-associated pancreatitis can be reduced: a randomized controlled trial. *Gastroenterology.* 2009;136(3):848–55. 10.1053/j.gastro.2008.11.044 19162029

[79] Nikkola J Räty S Laukkarinen J: Abstinence after first acute alcohol-associated pancreatitis protects against recurrent pancreatitis and minimizes the risk of pancreatic dysfunction. *Alcohol Alcohol.* 2013;48(4):483–6. 10.1093/alcalc/agt019 23538610

[80] Sadr-Azodi O Andrén-Sandberg Å, Orsini N: Cigarette smoking, smoking cessation and acute pancreatitis: a prospective population-based study. *Gut.* 2012;61(2):262–7. 10.1136/gutjnl-2011-300566 21836026

[81] da Costa DW Bouwense SA Schepers NJ: Same-admission versus interval cholecystectomy for mild gallstone pancreatitis (PONCHO): a multicentre randomised controlled trial. *Lancet.* 2015;386(10000):1261–8. 10.1016/S0140-6736(15)00274-3 26460661

[82] Siegel JH Veerappan A Cohen SA: Endoscopic sphincterotomy for biliary pancreatitis: an alternative to cholecystectomy in high-risk patients. *Gastrointest Endosc.* 1994;40(5):573–5. 10.1016/S0016-5107(94)70255-1 7988821

[83] Uomo G Manes G Laccetti M: Endoscopic sphincterotomy and recurrence of acute pancreatitis in gallstone patients considered unfit for surgery. *Pancreas.* 1997;14(1):28–31. 10.1097/00006676-199701000-00005 8981504

[84] Welbourn CR Beckly DE Eyre-Brook IA: Endoscopic sphincterotomy without cholecystectomy for gall stone pancreatitis. *Gut.* 1995;37(1):119–20. 10.1136/gut.37.1.119 7672659 PMC1382781

[85] Kochar B Akshintala VS Afghani E: Incidence, severity, and mortality of post-ERCP pancreatitis: a systematic review by using randomized, controlled trials. *Gastrointest Endosc.* 2015;81(1):143–149.e9. 10.1016/j.gie.2014.06.045 25088919

[86] Vadalà di Prampero SF Faleschini G Panic N: Endoscopic and pharmacological treatment for prophylaxis against postendoscopic retrograde cholangiopancreatography pancreatitis: a meta-analysis and systematic review. *Eur J Gastroenterol Hepatol.* 2016;28(12):1415–24. 10.1097/MEG.0000000000000734 27580214

[87] Kubiliun NM Adams MA Akshintala VS: Evaluation of Pharmacologic Prevention of Pancreatitis After Endoscopic Retrograde Cholangiopancreatography: A Systematic Review. *Clin Gastroenterol Hepatol.* 2015;13(7):1231–9; quiz e70-1. 10.1016/j.cgh.2014.11.038 25579870

[88] Wan J Ren Y Zhu Z: How to select patients and timing for rectal indomethacin to prevent post-ERCP pancreatitis: a systematic review and meta-analysis. *BMC Gastroenterol.* 2017;17(1):43. 10.1186/s12876-017-0599-4 28298192 PMC5353805

[89] Yang C Zhao Y Li W: Rectal nonsteroidal anti-inflammatory drugs administration is effective for the prevention of post-ERCP pancreatitis: An updated meta-analysis of randomized controlled trials. *Pancreatology.* 2017;17(5):681–8. 10.1016/j.pan.2017.07.008 28734720

[90] Luo H Zhao L Leung J: Routine pre-procedural rectal indometacin versus selective post-procedural rectal indometacin to prevent pancreatitis in patients undergoing endoscopic retrograde cholangiopancreatography: a multicentre, single-blinded, randomised controlled trial. *Lancet.* 2016;387(10035):2293–301. 10.1016/S0140-6736(16)30310-5 27133971

[91] Puig I Calvet X Baylina M: How and when should NSAIDs be used for preventing post-ERCP pancreatitis? A systematic review and meta-analysis. *PLoS One.* 2014;9(3):e92922. 10.1371/journal.pone.0092922 24675922 PMC3968039

[92] Cheon YK Cho KB Watkins JL: Efficacy of diclofenac in the prevention of post-ERCP pancreatitis in predominantly high-risk patients: a randomized double-blind prospective trial. *Gastrointest Endosc.* 2007;66(6):1126–32. 10.1016/j.gie.2007.04.012 18061712

[93] Ishiwatari H Urata T Yasuda I: No Benefit of Oral Diclofenac on Post-Endoscopic Retrograde Cholangiopancreatography Pancreatitis. *Dig Dis Sci.* 2016;61(11):3292–301. 10.1007/s10620-016-4251-x 27447477

[94] Kato K Shiba M Kakiya Y: Celecoxib Oral Administration for Prevention of Post-Endoscopic Retrograde Cholangiopancreatography Pancreatitis: A Randomized Prospective Trial. *Pancreas.* 2017;46(7):880–6. 10.1097/MPA.0000000000000852 28697127

[95] DiMagno MJ Wamsteker EJ Maratt J: Do larger periprocedural fluid volumes reduce the severity of post-endoscopic retrograde cholangiopancreatography pancreatitis? *Pancreas.* 2014;43(4):642–7. 10.1097/MPA.0000000000000101 24713841 PMC4024358

[96] Sagi SV Schmidt S Fogel E: Association of greater intravenous volume infusion with shorter hospitalization for patients with post-ERCP pancreatitis. *J Gastroenterol Hepatol.* 2014;29(6):1316–20. 10.1111/jgh.12511 24372871

[97] Choi JH Kim HJ Lee BU: Vigorous Periprocedural Hydration With Lactated Ringer's Solution Reduces the Risk of Pancreatitis After Retrograde Cholangiopancreatography in Hospitalized Patients. *Clin Gastroenterol Hepatol.* 2017;15(1):86–92.e1. 10.1016/j.cgh.2016.06.007 27311618

[98] Wu D Wan J Xia L: The Efficiency of Aggressive Hydration With Lactated Ringer Solution for the Prevention of Post-ERCP Pancreatitis: A Systematic Review and Meta-analysis. *J Clin Gastroenterol.* 2017;51(8):e68–e76. 10.1097/MCG.0000000000000856 28609383

[99] Zhang ZF Duan ZJ Wang LX: Aggressive Hydration With Lactated Ringer Solution in Prevention of Postendoscopic Retrograde Cholangiopancreatography Pancreatitis: A Meta-analysis of Randomized Controlled Trials. *J Clin Gastroenterol.* 2017;51(3):e17–e26. 28178088 10.1097/MCG.0000000000000781

[100] Park CH Paik WH Park ET: Aggressive intravenous hydration with lactated Ringer's solution for prevention of post-ERCP pancreatitis: a prospective randomized multicenter clinical trial. *Endoscopy.* 2018;50(4):378–85. 10.1055/s-0043-122386 29237204

[101] Mok SRS Ho HC Shah P: Lactated Ringer's solution in combination with rectal indomethacin for prevention of post-ERCP pancreatitis and readmission: a prospective randomized, double-blinded, placebo-controlled trial. *Gastrointest Endosc.* 2017;85(5):1005–13. 10.1016/j.gie.2016.10.033 27816497

[102] Smeets XJNM da Costa DW Fockens P: Fluid hydration to prevent post-ERCP pancreatitis in average- to high-risk patients receiving prophylactic rectal NSAIDs (FLUYT trial): study protocol for a randomized controlled trial. *Trials.* 2018;19(1):207. 10.1186/s13063-018-2583-x 29606135 PMC5879873

[103] Cao WL Yan WS Xiang XH: Prevention effect of allopurinol on post-endoscopic retrograde cholangiopancreatography pancreatitis: a meta-analysis of prospective randomized controlled trials. *PLoS One.* 2014;9(9):e107350. 10.1371/journal.pone.0107350 25202907 PMC4159328

[104] Kwanngern K Tiyapattanaputi P Wanitpukdeedecha M: Can a single dose corticosteroid reduce the incidence of post-ERCP pancreatitis? A randomized, prospective control study. *J Med Assoc Thai.* 2005;88 Suppl 4:S42–5. 16623000

[105] Sherman S Blaut U Watkins JL: Does prophylactic administration of corticosteroid reduce the risk and severity of post-ERCP pancreatitis: a randomized, prospective, multicenter study. *Gastrointest Endosc.* 2003;58(1):23–9. 10.1067/mge.2003.307 12838216

[106] Manolakopoulos S Avgerinos A Vlachogiannakos J: Octreotide versus hydrocortisone versus placebo in the prevention of post-ERCP pancreatitis: a multicenter randomized controlled trial. *Gastrointest Endosc.* 2002;55(4):470–5. 10.1067/mge.2002.122614 11923756

[107] Hu J Li PL Zhang T: Role of Somatostatin in Preventing Post-endoscopic Retrograde Cholangiopancreatography (ERCP) Pancreatitis: An Update Meta-analysis. *Front Pharmacol.* 2016;7:489. 10.3389/fphar.2016.00489 28018225 PMC5156829

[108] Li S Cao G Chen X: Low-dose heparin in the prevention of post endoscopic retrograde cholangiopancreatography pancreatitis: a systematic review and meta-analysis. *Eur J Gastroenterol Hepatol.* 2012;24(5):477–81. 10.1097/MEG.0b013e328351097f 22293331

[109] Shao LM Chen QY Chen MY: Nitroglycerin in the prevention of post-ERCP pancreatitis: a meta-analysis. *Dig Dis Sci.* 2010;55(1):1–7. 10.1007/s10620-008-0709-9 19160042

[110] Yuhara H Ogawa M Kawaguchi Y: Pharmacologic prophylaxis of post-endoscopic retrograde cholangiopancreatography pancreatitis: protease inhibitors and NSAIDs in a meta-analysis. *J Gastroenterol.* 2014;49(3):388–99. 10.1007/s00535-013-0834-x 23720090

